# A Reply to Contrary to the Conclusions Stated in the Paper, Only Dry Fat-Free Mass Was Different between Groups upon Reanalysis—Comment on: “Intermittent Energy Restriction Attenuates the Loss of Fat-Free Mass in Resistance Trained Individuals: A Randomized Controlled Trial”

**DOI:** 10.3390/jfmk5040086

**Published:** 2020-11-24

**Authors:** Bill I. Campbell

**Affiliations:** Exercise Science Program, Performance and Physique Enhancement Laboratory, University of South Florida, Tampa, FL 33620, USA; bcampbell@usf.edu

We would like to thank Dr. Sainsbury and colleagues for their interest in our publication, “Intermittent Energy Restriction Attenuates the Loss of Fat Free Mass in Resistance Trained Individuals: A Randomized Controlled Trial.” In their letter to the editor, the authors identified aspects related to the per protocol analysis and interpretation of the data presented from the investigation. We feel that their concerns are valid and fair, and given our dedication to open and transparent science, we wish to provide clarification and commentary on their concerns.

In relation to the per protocol analysis, it was stated that there was no report relative to an intention-to-treat analysis (ITT), which is correct. Our group had no intention of conducting an analysis of those participants who did not adhere to the diet and exercise program that was prescribed. On page 2 of the published manuscript, it states, “The primary purpose of this study was to compare body composition changes in resistance trained individuals after 7 weeks of either continuous energy restriction or intermittent restriction with a 2 days per week of carbohydrate refeeding during a supervised daily undulating resistance training program”. Furthermore, on page 6 of the published manuscript, the per protocol analysis is stated: “Data for all DVs were analyzed by a two group (refeed diet vs. continuous diet) x two time (pre-diet and post-diet) between–within factorial analysis of variance with repeated measures on the second factor via a per protocol analysis.” Given this stated purpose of the investigation and subsequent analysis, any interpretation of the study’s results is only in consideration for those who adhere to the prescribed diet and exercise program as described in the study protocol. Additionally, while ITT analyses are common in the realm of clinical nutrition, it is not the standard approach in the sports nutrition/exercise science literature and has not yet become common practice [1,2,3,4,5,6].

We appreciate that the authors of the letter to the editor took the raw data provided and conducted their own intention-to-treat analysis using the methodology they believed was best, formulating an interpretation of the data based on this analysis. The authors used a valid and defensible analytical approach, and their reported findings are in agreement with our analysis of the data with respect to the threshold for detecting significant differences (with a distinction in the magnitude of the differences between diet groups for dry fat-free mass (FFM)). There was also a re-analysis of the data provided in the publication’s online Supplementary Material using an analysis of covariance (ANCOVA) with baseline values as a covariate. Once again, this alternative analysis was in agreement with our initial analysis of the data. Specifically, the letter to the editor states “*Like* Campbell et al., we also found no significant difference between the intermittent/refeed and continuous diet groups for the change in FFM or RMR, albeit we did also observe significantly greater retention of dry FFM upon completion of the intermittent/refeed diet versus the continuous diet (*P* = 0.0004).” Our intention was to make our data set public for this reason—to allow for alternative analyses of the data, and to promote the widespread adoption of more rigorous standards for openness and transparency in the reporting of exercise science and kinesiology research. While we welcome secondary analysis of our data, it is worth noting that multiple researchers given the same data have been shown to report discrepant results based on key choices made in the analytical approach [7]. Transparent data sharing and secondary analyses should be celebrated and encouraged, but it is important to reinforce that secondary analyses are an informative practice to check the robustness of reported findings and provide an additional perspective, and some variability in reported effect estimates and p-values is to be expected given the large number of key decisions that go into any statistical analysis.

The other matter discussed was drawing conclusions based on nominal significance for fat-free mass (FFM) and resting metabolic rate (RMR). In addition to the two-way ANOVA analyses, effect sizes were calculated as was a reporting of 95% confidence intervals for each dependent variable of interest. Our interpretation of the data and study conclusions were in consideration of all of the data reported, and not exclusively restricted to the rejection of the null hypothesis at the 0.05 alpha level. While null hypothesis significance testing is indeed valuable for drawing inferences, it is important to recognize that the decision to exclusively interpret data based on the rejection of the null hypothesis at an alpha level of 0.05 is a widely accepted convention, but an imperfect convention nonetheless [8]. The authors are correct in their assertion that a statistically significant change in only one of the two groups does not necessarily imply that the groups displayed responses that were significantly divergent from one another. However, it is also important to recognize that the lack of a statistically significant interaction effect does not imply that the groups displayed statistically equivalent responses. In many cases, results from longitudinal studies with parallel groups will indicate that the groups did not have significantly divergent responses, however, did not have statistically equivalent responses. This is particularly common in small-sample research, in which effect estimates tend to be relatively imprecise. In such a scenario, there is insufficient evidence to reject the hypothesis that no effect exists in the population, and simultaneously, insufficient evidence to reject the hypothesis that a practically meaningful effect exists in the population.

Equivalence testing using the TOSTER package in R software, as described by Lakens and colleagues [9], indicates that the observed FFM and RMR data in our study show that the two groups were not statistically different from one another, but not statistically equivalent. The data provided insufficient evidence to reject the null hypothesis of no effect in this population, but also provided insufficient evidence to reject the hypothesis of a practically meaningful effect in this population. In light of these considerations, and the relative consequences of committing type I versus type II errors for the given research question, we feel it is informative to interpret raw values, effect sizes, and confidence intervals in conjunction with traditional null hypothesis testing. Our inferences regarding FFM and RMR were based on the interpretation of the totality of the evidence, but we apologize if the manuscript unintentionally implied that we observed changes that represented a statistically significant between-group difference at an alpha level of 0.05. It is also worth noting that the direction of between-group differences for all three dependent variables of interest (FFM, RMR, and dry FFM) were generally consistent in reflecting modestly more favorable changes for the refeed group than the continuous group. Given the close theoretical relationship between all three variables, the fact that all three changed with similar directionality provides some weak evidence that the patterns observed are unlikely to be attributable to measurement error or random variability alone.

Our summary of the investigation is stated in three places. In the abstract (page 1), our statement reads: “A 2-day carbohydrate refeed preserves FFM, dryFFM, and RMR during energy restriction compared to continuous energy restriction in RT-individuals.” In the discussion (page 8), our statement reads: “The primary finding of this study is that a consecutive 2-day carbohydrate refeed preserves fat-free mass/dry fat-free mass during an energy-restricted diet as compared to continuous energy restriction in a population that prioritizes the maintenance of muscle mass when dieting. A secondary finding was that resting metabolic rate was better maintained, albeit slightly, with the 2-day carbohydrate refeed.” Similarly, on page 10 in the study conclusions, our statement reads: “In summary, this is the first investigation, to our knowledge, to demonstrate a preservation of fat-free mass and resting metabolic rate in response to a 2-day carbohydrate refeed during an energy restricted diet in lean, resistance trained males and females.”

With respect to ensuring that the conclusions that we make can be supported by the analysis of the data, and to eliminate confusion as to the between-group differences observed in our investigation, we believe a better conclusion is: “A 2-day carbohydrate refeed preserves dry fat-free mass. The changes in fat-free mass and RMR were not significantly different between the two diet treatments, although it bears noting that both fat-free mass and RMR were more effectively maintained in the group of subjects who were given 2-day refeeds, despite having the same weekly average deficit as the group who dieted continuously.”

Interestingly, when reading the published literature within the very small scope of non-linear dieting, it becomes readily apparent that other respected research groups report similar findings (non-significant between-group changes while observing within-group changes in only one group over time) to those reported by our group in the current study. A sampling of the most recent publications in this area (within the past 5 years only) demonstrates similar approaches in reporting such analyses in both relevant primary and secondary research variables under discussion presently, including body weight [10], resting metabolic rate [11], respiratory quotient [12], and % fat mass/% lean mass [13].

We would like to extend our gratitude to Sainsbury and colleagues for their thoughtful inquiries regarding our research. It is our hope that other research groups will attempt to replicate our methodological design in resistance trained individuals under similar quality-control parameters—namely the direct supervision of the resistance exercise workouts and the daily tracking of caloric and macronutrient intake throughout the study duration. We also hope it will become more common for exercise science and sports nutrition researchers to make their raw data publicly available as we have, to promote greater transparency and more informative discourse within the field.
